# Sphere Culture of Murine Lung Cancer Cell Lines Are Enriched with Cancer Initiating Cells

**DOI:** 10.1371/journal.pone.0049752

**Published:** 2012-11-13

**Authors:** Brian J. Morrison, Jason C. Steel, John C. Morris

**Affiliations:** Division of Hematology-Oncology, Department of Medicine, University of Cincinnati, Cincinnati, Ohio, United States of America; University Magna Graecia, Italy

## Abstract

Cancer initiating cells (CICs) represent a unique cell population essential for the maintenance and growth of tumors. Most *in vivo* studies of CICs utilize human tumor xenografts in immunodeficient mice. These models provide limited information on the interaction of CICs with the host immune system and are of limited value in assessing therapies targeting CICs, especially immune-based therapies. To assess this, a syngeneic cancer model is needed. We examined the sphere-forming capacity of thirteen murine lung cancer cell lines and identified TC-1 and a metastatic subclone of Lewis lung carcinoma (HM-LLC) as cell lines that readily formed and maintained spheres over multiple passages. TC-1 tumorspheres were not enriched for expression of CD133 or CD44, putative CIC markers, nor did they demonstrate Hoechst 33342 side population staining or Aldefluor activity compared to adherent TC-1 cells. However, in tumorsphere culture, these cells exhibited self-renewal and long-term symmetric division capacity and expressed more Oct-4 compared to adherent cells. HM-LLC sphere-derived cells exhibited increased Oct-4, CD133, and CD44 expression, demonstrated a Hoechst 33342 side population and Aldefluor activity compared to adherent cells or a low metastatic subclone of LLC (LM-LLC). In syngeneic mice, HM-LLC sphere-derived cells required fewer cells to initiate tumorigenesis compared to adherent or LM-LLC cells. Similarly TC-1 sphere-derived cells were more tumorigenic than adherent cells in syngeneic mice. In contrast, in immunocompromised mice, less than 500 sphere or adherent TC-1 cells and less than 1,000 sphere or adherent LLC cells were required to initiate a tumor. We suggest that no single phenotypic marker can identify CICs in murine lung cancer cell lines. Tumorsphere culture may provide an alternative approach to identify and enrich for murine lung CICs. Furthermore, we propose that assessing tumorigenicity of murine lung CICs in syngeneic mice better models the interaction of CICs with the host immune system.

## Introduction

Lung cancer is the cause of almost 20% of all cancer deaths in the United States [1]. It accounts for more deaths than the next four most common cancers combined. Despite improvements in diagnosis and treatment, the overall 5-year survival for all patients with lung cancer is a dismal 15%, and this declines to less than 2% in patients with metastatic disease [2]. Hence, lung cancer is a major public health problem, and a better understanding of the disease biology and improvements in treatment are greatly needed.

Increasing evidence supports the concept of a specialized population of cells within tumors termed cancer initiating cells (CICs), alternatively “cancer stem cells” or tumor-initiating cells. These cells are thought to be responsible for the tumor’s origin, maintenance, progression, and resistance to therapy. CICs display functional characteristics of stem cells such as the capacity for self-renewal and the ability to give rise to differentiated progeny. Putative CICs have been identified in myeloid leukemia [3], , and tumors of the brain [5], [6] and breast [7]. More recently, lung CICs have been isolated from human cell lines and patient samples [8]–[15]. Investigating lung CICs may increase understanding of the origin of lung cancer and may lead to novel therapeutic approaches targeting these cells.

CICs have been shown to form multicellular three-dimensional spheres *in vitro* when grown in non-adherent serum-free conditions [6], [16]. Under these conditions, most tumor cells undergo anoikis, a form of programmed cell death, whereas the rare CICs divide to generate tumor spheroids. The sphere assay and a recently proposed mathematical interpretation allow for the assessment of the symmetric division expansion rate of malignant stem cell-like cells, and the evaluation of the effects of treatment on the self-renewal and proliferative activity of these cells [17]. This assay is a powerful tool to assess the functional and phenotypic properties associated with CICs. In a study using patient lung cancer samples, sphere formation was found in seven of 19 tumors examined [10]. These cells were found to have *in vitro* and *in vivo* properties of CICs including self-renewal, proliferative and differentiation capacity, expression of CD133, enrichment for side population (SP) cells, expression of the embryonic “stemness” genes Oct-4 and NANOG, chemotherapy resistance, and tumorigenicity. Sphere culture has been used to enrich for lung cancer CIC populations [8], [9], [13], [14].

**Figure 1:**
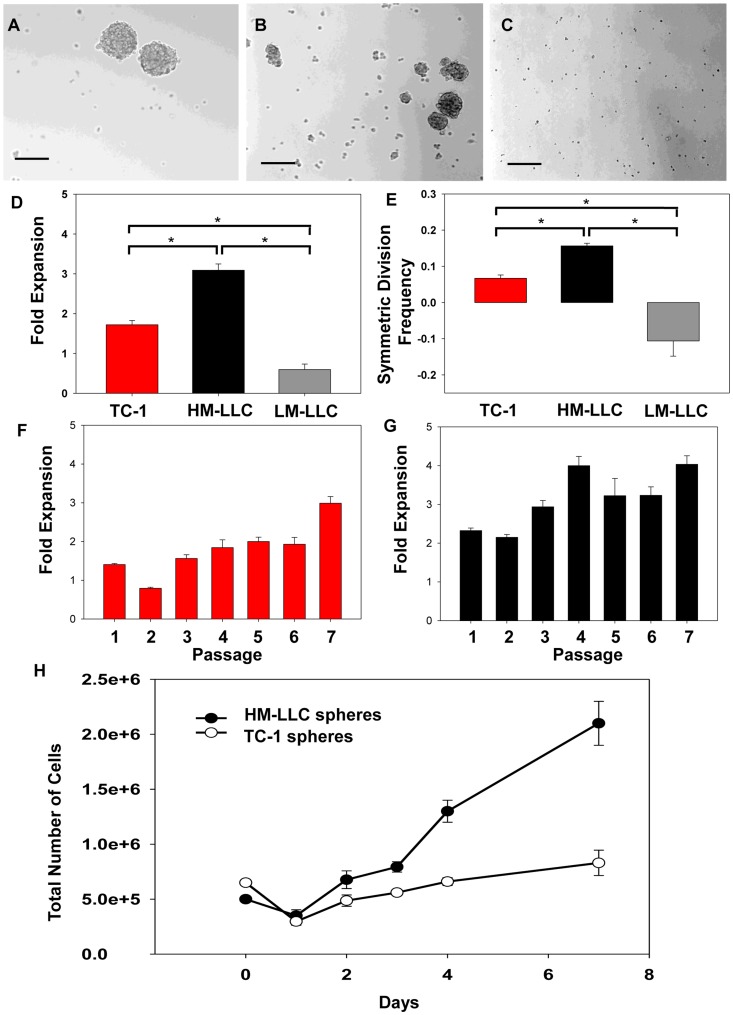
*In vitro* tumorsphere assays. Photomicrographs (10X) of passage 2 day 7 sphere cultures of (**A**) TC-1 spheres, (**B**) HM-LLC spheres and (**C**) LM-LLC sphere media culture. Bar = 100 µm. (**D**) Total fold expansion and (**E**) symmetric division frequency over eight passages for TC-1 spheres (red) and HM-LLC spheres (black), and two passages for LM-LLC sphere media culture (grey) shows significant differences; *P<0.001. (**F–G**) Fold expansion over seven passages for TC-1 spheres (red) and HM-LLC spheres (black). (**H**) Passage 2 spheres were cultured and counted for total number of cells on the days as shown. Experiment repeated twice, representative results shown. On day 1 the total number of cells in culture is reduced. The surviving mitogen-responsive cancer initiating cells are responsible for cell proliferation and generating fresh multi-cellular spheres that can be harvested on day 7. Bars: ±Standard error of the mean (SEM).

**Table 1:** Murine lung cancer cell lines sphere forming capacity.

Cell Line	Sphere Forming Capacity*	Long Term Proliferation Assay†	Fold Expansion§
TC-1	+	+	1.7±0.7
LLC (ATCC)	−		
HM-LLC	+	+	3.1±0.8
LM-LLC	−		
18948	+	**−**	0.4±0.3
18955-Floating	−		
18955-Adherent	+	−	0.2±0.1
18959	+	−	0.4±0.6
MLE12	+	−	0.8±0.5
MLE15	+	−	1±0.5
393P	−		
344P	+	−	0.8±0.2
344SQ	+	−	0.9±0.4

* Ability to form three-dimensional multicellular spheroids.

† Mean fold expansion over 1 for serial passage, and ability to passage as spheres more than 3 passages.

§ Fold expansion average ±standard deviation.

Murine lung cancer has not been well characterized for the presence and frequency of CICs. The majority of studies of lung CICs have used human cell lines or primary tumor samples, with tumorigenicity examined solely in immunocompromised mouse models. Importantly, interactions between the immune system and the tumor microenvironment have been demonstrated to play a role in the response to therapy. Indeed, lung cancer and many other tumors occurring in the immunocompromised host behave more aggressively, respond poorly to treatment, and have poorer outcomes [18], [19]. Therefore, to more accurately model the immune interactions involved in the establishment of tumors by CICs, we investigated murine lung tumor cells enriched for CICs in an immunocompetent syngeneic animal. The current study reports the identification and characterization of murine lung CICs using sphere culture methods and the comparative assessment of these cells in syngeneic and immunocompromised mouse models.

## Materials and Methods

### Cells

Murine TC-1 and parental Lewis lung carcinoma (LLC) cells were purchased from ATCC (Manassas, VA). The high metastatic (HM-LLC) and low metastatic (LM-LLC) subclones of LLC [20], [21] were gifts of Dr. Zhongyun Dong (University of Cincinnati, Cincinnati, OH), and were cultured as adherent cells in Dulbecco’s modified Eagle media (DMEM) (Mediatech, Inc., Manassas, VA) supplemented with 10% fetal calf serum (FCS) (Invitrogen, Carlsbad, CA) and 1% penicillin G-streptomycin (Invitrogen). Cell lines 18948, 18955-Adherent, 18955-Floating (a non-adherent clone of 18955 cells), and 18959 cells are *de novo* murine small cell lung cancer (SCLC) cell lines and were a gift of Dr. Kathryn Wikenheiser-Brokamp (Cincinnati Children’s Medical Center, Cincinnati, OH). These cell lines were studied under The University of Cincinnati Institutional Animal Care and Use Committee approval (Protocol No. 11-03-21-01). Murine lung tumor cell lines MLE12 and MLE15 [22], derived from transgenic mice harboring the simian virus 40 (SV40) large tumor antigen under transcriptional control of the human surfactant protein C (SP-C) promoter, were gifts of Dr. Jeffrey Whitsett (Cincinnati Children’s Medical Center). SCLC cell lines and MLE12 and MLE15 cells were cultured in HITES media [23]. Murine adenocarcinoma lung cancer cell lines 393P, 344P, and 344SQ [24] derived from Kras^La1/+^p53^R172HΔG^ mice were a gift of Jonathan M. Kurie (The University of Texas, MD Anderson Cancer Center, Houston, TX) and were cultured in DMEM with 10% FCS.

### Animals and Ethics Statement

C57Bl/6 mice were obtained from Jackson Laboratories (Bar Harbor, ME) and NOD-SCIDγ (NSG, NOD.Cg-*Prkdc^scid^ Il2rg^tm1Wjl^*/SzJ) mice from an established breeding colony at Cincinnati Children’s Medical Center. The University of Cincinnati Institutional Animal Care and Use Committee granted approval for this work (Protocol No. 11-03-21-01). All procedures were in accordance with institutional animal welfare guidelines, and all efforts were made to minimize suffering.

**Figure 2:**
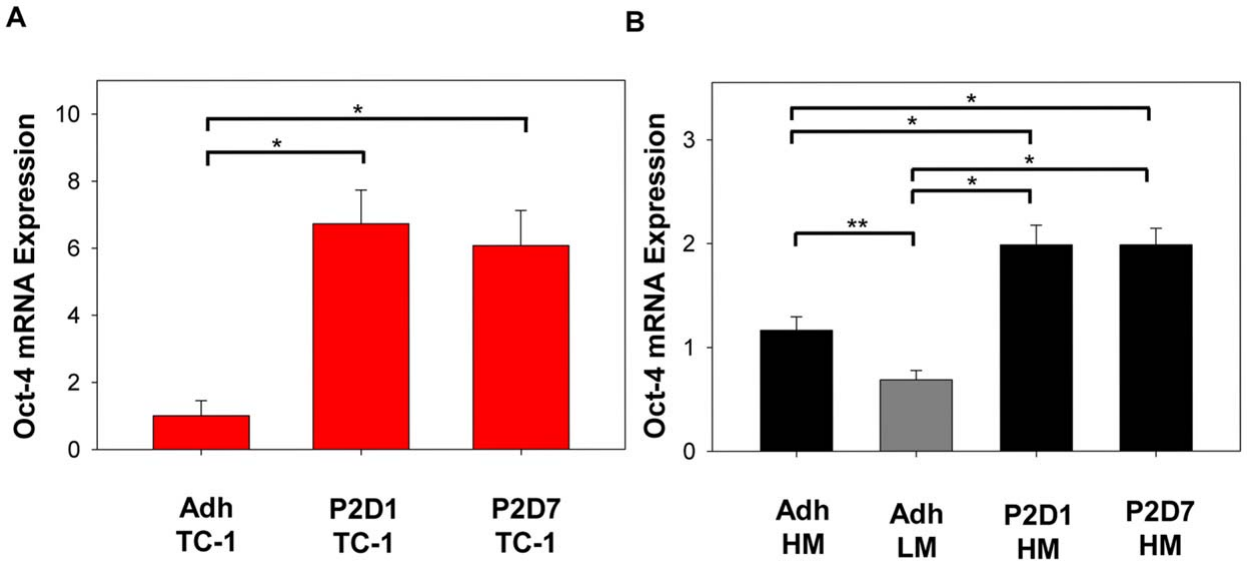
Oct-4 expression is increased in spheres compared to adherent cells. Real time PCR quantitation was done for Oct-4, a gene associated with stem cell self-renewal, mRNA expression. TC-1 spheres (**A**) and HM-LLC spheres (**B**) demonstrate increased Oct-4 expression compared to matched adherent cells; *P≤0.001. HM-LLC cells expressed significantly more Oct-4 than LM-LLC adherent cells; **P = 0.005. Plots represent combined data from 2–4 biological replicates, each with three technical replicates. TC-1 cells (red); LM-LLC adherent (grey); HM-LLC cells (black). Bars: ±SEM.

**Figure 3:**
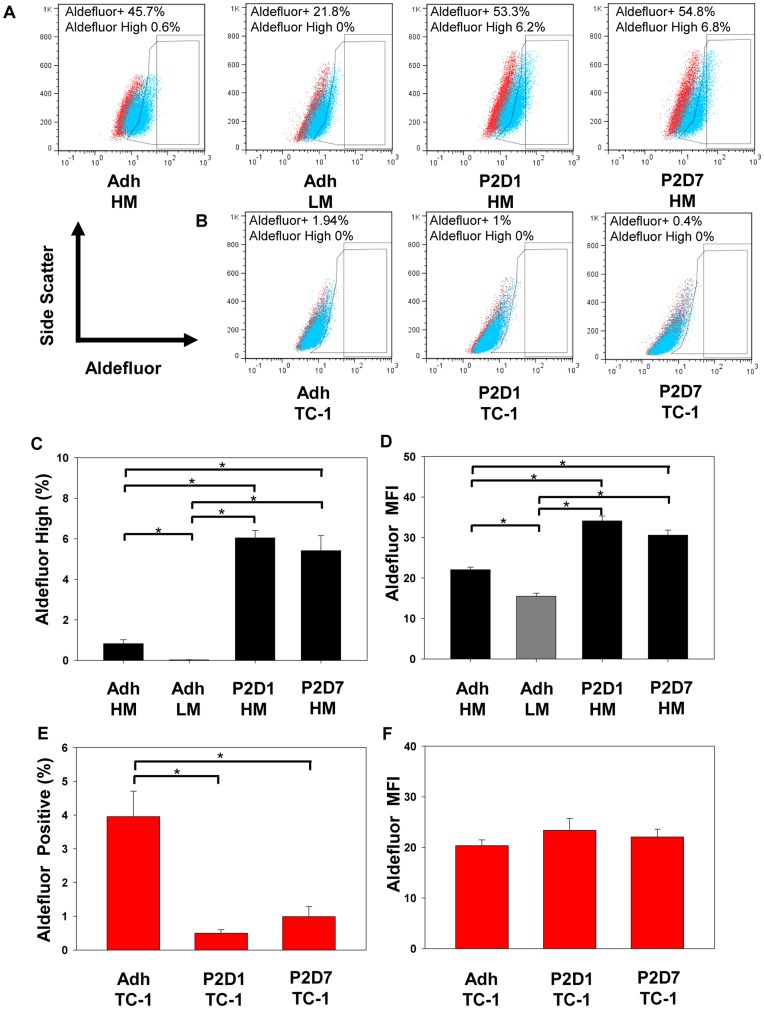
Aldefluor expression is enhanced in HM-LLC but not in TC-1 spheres compared to adherent cells. (**A–B**) Representative plots showing Aldefluor staining (blue) and DEAB control staining (red) for HM-LLC, LM-LLC and TC-1 adherent cells compared to passage 2, day 1 and day 7 sphere-derived cells. Aldefluor high expressing cells (rectangular box) are significantly greater in (**C**) frequency and (**D**) mean fluorescent intensity (MFI) of Aldefluor within HM-LLC spheres compared to LM-LLC or HM-LLC adherent cells, and HM-LLC adherent cells express more Aldefluor than LM-LLC adherent cells; frequency (**C**) *P≤0.001; MFI (**D**) *P≤0.002. Compared to DEAB staining, Aldefluor positivity (non-rectangular box) was found to be significantly higher in TC-1 adherent cells compared to spheres (**E**); *P≤0.004. MFI of Aldefluor within the TC-1 groups (**F**) was not found to be significantly different. No Aldefluor high population was noted for TC-1 spheres or adherent cells. Plots represent combined data from two biological repeats with three technical replicates each. Bars: ±SEM.

**Figure 4:**
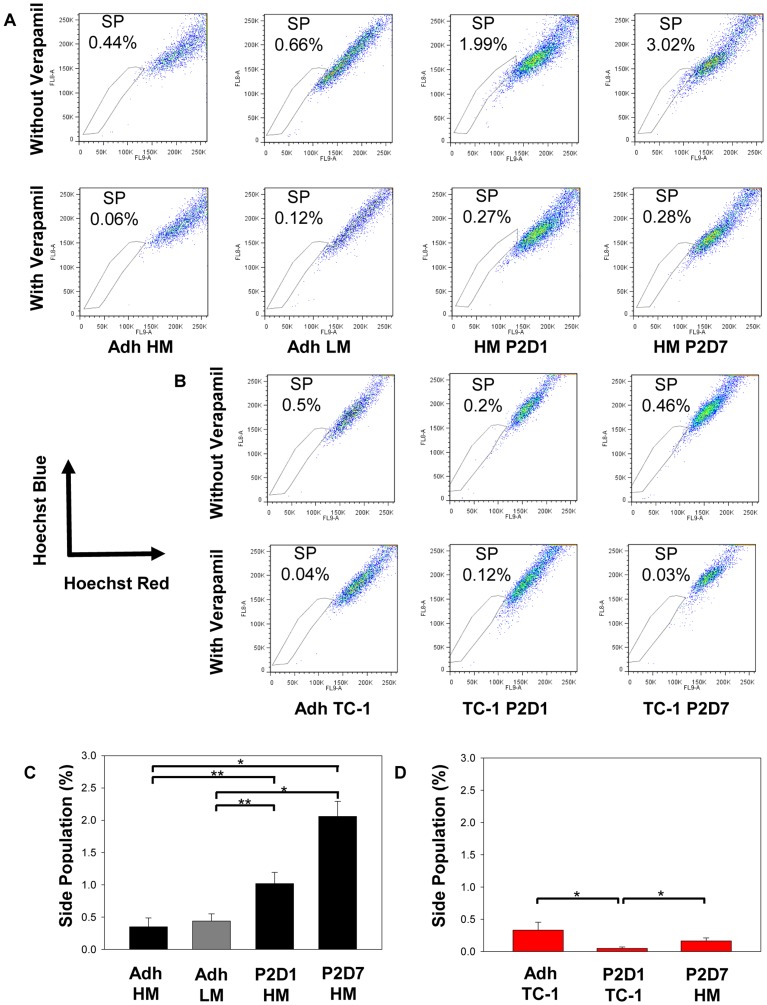
Hoechst 33342 side population (SP) expression is enhanced in HM-LLC but not in TC-1 spheres compared to adherent cells. Representative SP plots for (**A**) LLC cells and (**B**) TC-1 cells comparing matched adherent cells to passage 2, day 1 and day 7 sphere-derived cells. Hoechst 33342 staining was confirmed with verapamil blockade. (**C**) SP frequency was significantly increased in HM-LLC spheres compared to adherent cells; *P<0.001; **P≤0.012. HM-LLC and LM-LLC adherent cells were not significantly different for SP frequency. (**D**) SP was not found to be upregulated in sphere-derived TC-1 cells compared to matched adherent cells. Day 1 sphere-derived cells had a significantly lower frequency of SP cells compared to adherent cells and day 7 sphere-derived cells; *P = 0.03. Plots represent combined data from three biological replicates with three technical replicates each. Bars: ±SEM.

**Figure 5:**
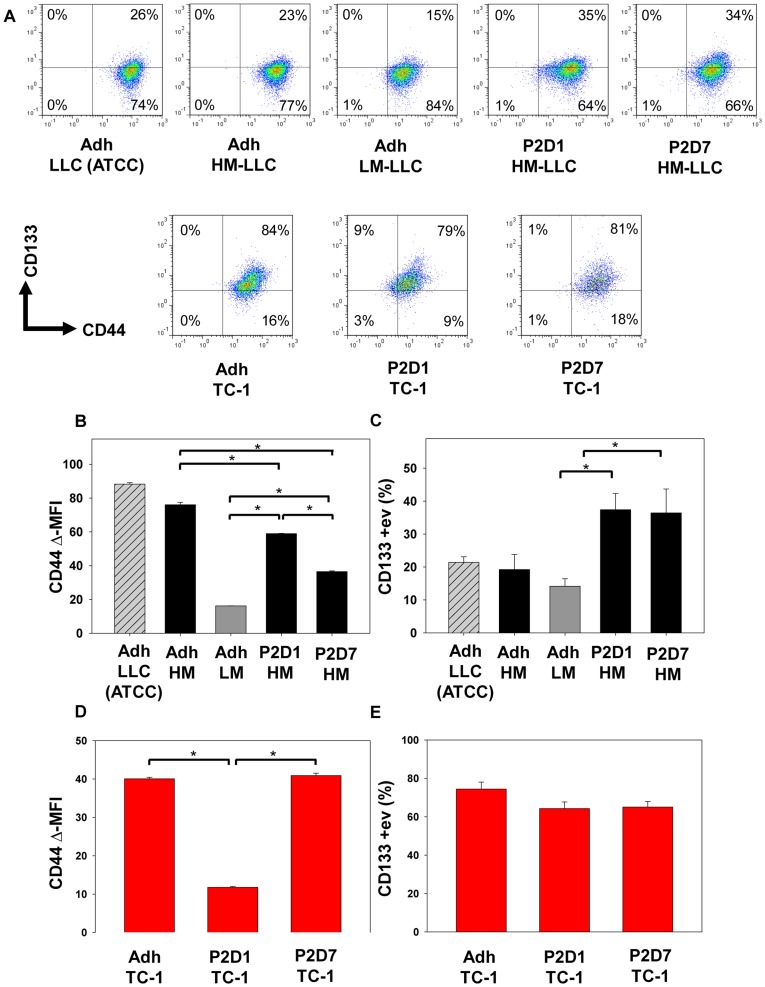
CD133/CD44 cell surface expression. (**A**) Representative flow cytometry plots for CD133 (Y-axis) and CD44 (X-axis) co-staining. (**B and D**) The mean fluorescent intensity (MFI) of CD44 is shown corrected for intensity of isotype staining for LLC and TC-1 cells. (**B**) HM-LLC spheres and adherent cells as well as LLC (ATCC) cells express higher amounts of CD44 compared to LM-LLC cells. Sphere-derived cells were found to have a lower MFI for CD44 compared to HM-LLC adherent cells. D7 sphere-derived cells had a lower MFI for CD44 compared to D1 cells; *P<0.001. **(D**) Day 1 TC-1 sphere-derived cells express less CD44 than day 7 or adherent cells; *P≤0.002. (**C**) CD133 frequency of positive cells is higher in HM-LLC spheres compared to LM-LLC adherent cells; *P≤0.043. CD133 expression was not significantly different between HM-LLC adherent cells and spheres or amongst the three different LLC adherent cells. (**E**) No significant difference in CD133 expression amongst the TC-1 culture conditions. Experiment done in triplicate, representative results shown. Bars: ±SEM.

**Figure 6:**
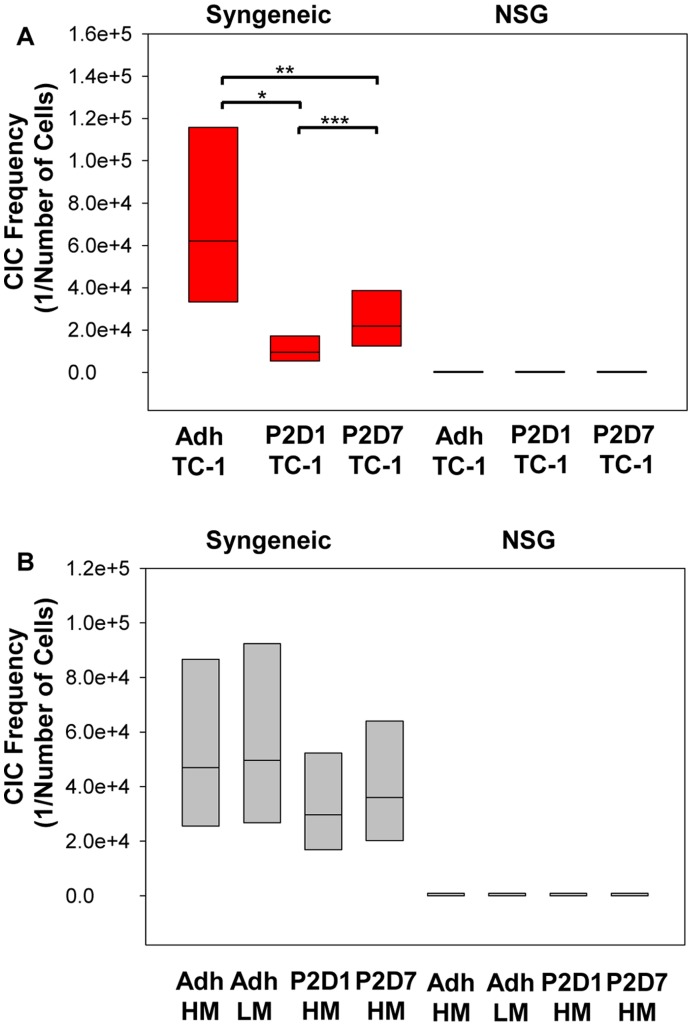
*In vivo* tumorigenicity of TC-1 and LLC cells in immunocompetent and immunodeficient mice. Confidence interval plot of limiting dilution analysis is shown with the y-axis displaying the estimated frequency of cancer initiating cells (CIC). (**A**) A limiting dilution of TC-1 cells (120,000, 80,000, 40,000, 10,000, 5,000, and 1,000) were injected subcutaneously into syngeneic C57BL/6 mice (n = 4 to 8). Sphere derived cells were found to be more tumorigenic than adherent cells; *P≤0.001; **P<0.02. Day 1 sphere-derived cells were more tumorigenic than day 7; ***P = 0.04. A limiting dilution (10,000, 1,000, and 500 cells) (n = 6 to 8) of the same three groups were injected into NSG mice and CIC frequency was found to be below 1/500 for all groups. (**B**) Analysis of LLC limiting dilution tumor experiment for syngeneic (80,000, 40,000, 20,000, and 10,000 cells) and immunocompromised (NSG) mice (80,000, 40,000, 10,000, and 1,000 cells) (n = 4 to 9). No significant differences noted; however, sphere-derived cells trended towards being more tumorigenic than HM or LM adherent cells in C57BL/6 mice. Lower numbers of implanted cells were needed to initiate tumors in immunocompromised animals than in syngeneic animals with the estimated CIC frequency below 1/1000 for all LLC groups.

### Tumorsphere Assays

Cells were cultured as tumorspheres in DMEM/F12 (Hyclone Laboratories, Inc., Logan, UT) containing 20 ng/mL rhEGF (R&D Systems, Minneapolis, MN), 10 ng/mL rhbFGF (R&D Systems), 4 µg/mL heparin sulfate (Sigma, St. Louis, MO), 0.15% bovine serum albumin (Sigma), and 1% penicillin G-streptomycin [25]. Sphere forming capacity (SFC) was determined by the ability to form three-dimensional spheroids in culture over a period of five to nine days. For assessment of long term proliferation and fold expansion (FE), spheres were disassociated and passaged every 7 days using 0.05% trypsin-EDTA (Invitrogen). Cells were grown at 37°C in 5% CO_2_. Sphere cultures were grown in low-adherent flasks (Nunc, Penfield, NY). Cells were counted using a Countess™ (Invitrogen) automated cell counter. Fold expansion and symmetric division frequency [17] were assessed for each cell line, as well as the ability for the cells to culture as spheroids for greater than three passages.

### Cell Surface Staining and Aldefluor Assay

For surface marker analysis, phycoerythrin (PE)-labeled anti-mouse CD133 (clone 13A4, eBioscience, Inc. San Diego, CA) was used at a 1∶33 dilution and fluorescein isothiocyanate (FITC)-labeled anti-human/mouse CD44 (clone IM7, eBioscience) was used at a 1∶100 dilution. FITC-labeled rat IgG2bκ was used as an isotype control (BD Biosciences, Franklin Lakes, NJ). An Aldefluor assay kit was used to detect the enzyme aldehyde dehydrogenase 1 (ALDH1; STEMCELL Technologies, Inc., Vancouver, BC). Briefly, cells were incubated for 60 minutes at 37°C in the presence of Aldefluor reagent, alone or with the addition of a diethylaminobenzaldehyde (DEAB)-containing solution that blocks ALDH1 activity. Data was collected using an Epics XL-MCL flow cytometer (Beckman Coulter, Inc., Brea, CA). Non-viable cells were excluded from analysis using propidium iodide (Sigma) staining. Activity was assessed using matching DEAB control samples. Data was analyzed using FlowJo software (Tree Star, Inc., Ashland, OR).

### Hoechst 33342 side Population (SP)

Hoechst 33342 dye staining SP analysis was conducted using methods adapted from Goodell et al. [26]. Briefly, one million cells were incubated with 10 µg/mL Hoechst 33342 (Sigma) for 90 minutes at 37°C. Confirmation of SP cells was conducted using 50 µM verapamil (Sigma) to block the efflux of the dye. Data was collected using a LSRII flow cytometer (BD Biosciences). Non-viable cells were excluded from analysis.

### Real-time Reverse Transcription-polymerase Chain Reaction (RT-PCR)

Total RNA was extracted using PureLink™ RNA mini-kit (Invitrogen). RNA was reverse transcribed in 20 µL using the Verso cDNA kit (Thermo Fisher Scientific, Surrey, UK) and the GeneAmp PCR System 9700 thermocycler (Applied Biosystems, Foster City, CA). Analysis of Oct-4 expression was carried out using Plexor® qPCR System (Promega, Madison, WI) reagents and StemElite™ Mus-Pou5f1/Actb primer pairs (Promega) containing primers for both Oct-4 and the β-actin gene. Data was collected using the Bio-Rad CFX96™ RT-System (Bio-Rad Laboratories, Hercules, CA) and analyzed using Plexor® analysis software. All real-time RT-PCR results were compiled using three to six technical repeats for each biological replicate and two to four biological repeats were done for each sample – matched adherent cells and passage 2 (P2) day 1 (D1) and day 7 (D7) sphere-derived cells. Data was normalized to endogenous β-actin for each sample. Samples were standardized to either TC-1 or HM-LLC adherent cells to compare expression levels among samples.

### In vivo Tumor Initiation

A limiting dilution of TC-1 (120,000, 80,000, 40,000, 10,000, 5,000, or 1,000) cells from matched adherent and P2 sphere-derived D1 or D7 cultures were subcutaneously injected into the flanks of C57Bl/6 mice (n = 4 to 8). Another limiting dilution of cells (10,000, 1,000, and 500 cells) for the same three conditions were also injected into NSG mice (n = 6 to 8). Similarly, for LLC cells a limiting dilution experiment of 80,000, 40,000, 20,000, or 10,000 cells for C57Bl/6 and 80,000, 40,000, 10,000, or 1,000 cells for NSG mice was conducted (n = 4 to 9). Mice were monitored twice weekly for time-to-tumor formation. Tumor-free survival up to day 65 was assessed.

### Statistical Analysis

SigmaPlot 11.0 (Systat Software, Inc., Chicago, IL) was used for statistical analysis. Student’s *t*-test was used to compare groups. For *in vivo* tumor formation, limiting dilution analysis estimating differences in stem cell/cancer initiating cell frequency among samples was conducted using Extreme Limiting Dilution Analysis **(**ELDA) software as previously described [27]. The level of statistical significance was set at 0.05.

## Results

### Sphere Forming Capacity

SFC was examined in 13 murine lung cancer cell lines or subclones of cell lines derived from various murine genetic backgrounds (**Table 1**). SFC was noted for nine of these cell lines. However, SFC did not always correlate with the ability to culture as spheres over serial passages. Of the nine cell lines that formed spheres and were tested for long term culture capacity, only two, TC-1 and HM-LLC demonstrated sustained growth over serial passages. TC-1 and HM-LLC cells were capable of forming three-dimensional spheroids in culture (**Figures 1A and 1B**). LM-LLC cells (**Figure 1C**) and parental LLC cells did not form spheres and were incapable of prolonged passage in sphere culture. TC-1 and HM-LLC spheres exhibited a FE potential >1 over multiple passages (**Figure 1D**). Sphere cultures of TC-1 and HM-LLC exhibited a positive cancer stem cell/CIC symmetric division frequency, whereas LM-LLC cells did not (**Figure 1E**). This indicates that sphere media culture conditions can identify putative CICs for these two cell lines. FE was relatively stable over passage number for TC-1 (**Figure 1F** **)** and HM-LLC (**Figure 1G**) spheres. Within 24 hours of passage approximately 30% (HM-LLC) or 54% (TC-1) cells undergo anoikis/cell death (**Figure 1H**). The remaining mitogen-responsive cells at D1 went on to form spheres by D7. We hypothesized that D1 cells were enriched for CICs compared to D7 cells, as the D7 cells were more likely to be composed of some CICs and greater numbers of differentiated tumor cells.

### Oct-4 mRNA Expression is Enhanced in Sphere-derived Cells

Expression of Oct-4, a gene involved in stem cell self-renewal and a key regulator of pluripotency that can alone reprogram cells to pluripotency [28], was assessed in matched adherent and passage 2, D1 and D7 sphere cells for TC-1 and HM-LLC, and adherent LM-LLC cells. Data was normalized to expression of β-actin and standardized to expression levels of either TC-1 (**Figure 2A**) or HM-LLC adherent cells (**Figure 2B**). Sphere-derived cells expressed significantly greater Oct-4 mRNA than matched adherent cells; P≤0.001. TC-1 sphere cultured cells from either D1 or D7 expressed approximately 6-fold more Oct-4 compared to adherent cells. HM-LLC spheres from either D1 or D7 expressed over 1.7 times more Oct-4 than adherent cells. LM-LLC adherent cells expressed Oct-4 at approximately 60% the levels of adherent HM-LLC cells. Expression of SOX2 and NANOG that are associated with self-renewal and stemness, were not found to be significantly differently expressed between spheres and adherent cells (data not shown).

### Aldefluor Activity is Enhanced in HM-LLC but not in TC-1 Spheres

ALDH1 expression and activity has been correlated with CIC activity and increased aggressiveness of lung tumors [12]. We examined ALDH1 activity in matched adherent and sphere cultures. Representative plots are shown for adherent HM-LLC and LM-LLC compared to P2, D1 and D7 sphere cultured cells (**Figure 3A**) and for adherent TC-1 cells compared to P2, D1 and D7 sphere cultured cells (**Figure 3B**). For HM-LLC spheres, a subset of high Aldefluor expressing cells were found to be significantly increased in sphere cultured cells compared to HM-LLC or LM-LLC adherent cells (**Figure 3A**); P≤0.001 for frequency, and P≤0.002 for mean fluorescent intensity (MFI) (**Figure 3C and 3D**). TC-1 adherent and sphere-derived cells expressed low levels of Aldefluor activity (**Figure 3B**). Adherent TC-1 cells demonstrated more Aldefluor-positive cells compared to sphere-derived cells (**Figure 3E**); P≤0.004. These levels were not significantly different when analyzed for MFI (**Figure 3F**).

### SP is Enhanced in HM-LLC but not in TC-1 Spheres

We examined matched adherent cells, and P2, D1 and D7 sphere cultures with Hoechst 33342 for SP. As a control, verapamil was used to disrupt the activity of the Hoechst 33342 transporter. SP was defined as the diminished population of cells in the presence of verapamil. Examples of profiles for LLC cells (**Figure 4A**) and TC-1 cells (**Figure 4B**) are shown. SP frequency was found to be increased within spheres of HM-LLC compared to adherent cells of either HM-LLC or LM-LLC; P≤0.012 for D1 spheres and P<0.001 for D7 spheres (**Figure 4C**). The average fraction of SP cells for three biological repeats for HM-LLC P2, D1 spheres was 1±0.5%, for P2, D7 spheres 2±0.7%, for HM-LLC adherent cells 0.4±0.4% and for LM-LLC adherent cells 0.4±0.3%. TC-1 adherent and sphere-cultures expressed low frequencies of SP cells. SP frequency was not found to be enhanced for sphere cultured compared to adherent TC-1 (**Figure 4D**). Day 1 sphere-derived cells were found to have a decrease in frequency of SP cells compared to adherent cells or D7 sphere-derived cells; P = 0.03.

### CD133 Expression is Increased in HM-LLC Spheres and CD44 Expression is Enhanced in HM-LLC Cells Compared to LM-LLC Cells

In addition to assessing functional characteristics of CICs such as ALDH1 activity and Hoechst 33342 dye efflux, we also sought to characterize TC-1 and LLC cells for the expression of CD44 and CD133, putative CIC cell surface markers. Representative plots for CD44 and CD133 are shown (**Figure 5A**). Among TC-1 and LLC cells, all of the cells expressed CD44. CD44 expression for HM-LLC spheres compared to HM-LLC adherent cells demonstrated a decrease for spheres in overall expression (**Figures 5A and 5B**). CD44 expression was variable among the LLC cell lines and culture conditions (**Figures 5A and 5B**). Adherent cell lines displayed a more homogenous expression of CD44, whereas sphere-cultured cells exhibited a more heterogenous expression with some cells expressing decreased amounts of CD44 while most remained CD44 bright. CD44 MFI expression was found to decrease between D1 and D7 cells; P<0.001 (**Figure 5B**). LM-LLC cells displayed the least intense staining for CD44 compared to all other LLC cell types and culture conditions; P<0.001 (**Figure 5A**). No difference was detected for expression of CD133 among HM-LLC adherent cells and P2, D1 and D7 sphere culture cells; however, there was a significant increase in the frequency of CD133 positivity among HM-LLC spheres, both D1 and D7 cultured cells compared to LM-LLC cells (**Figure 5C**).

CD44 expression was decreased in P2, D1 sphere-derived cells for TC-1 cells compared to adherent cells and P2, D7 cells (**Figure 5D**). No difference in CD44 expression was noted for P2, D7 TC-1 cells compared to adherent TC-1 cells. No significant differences were noted in expression of CD133 among the TC-1 cell culture conditions (**Figure 5E**).

### Sphere-derived Cells Exhibit Greater Tumorigenicity in Syngeneic Mice Compared to Adherent Cells

We assessed the tumorigenicity of D1 and D7 spheres compared to adherent cells. We also examined the tumorigenicity of these cells in immunocompetent syngeneic mice compared to immunocompromised NSG mice in order to better address the CIC model in these two different mouse environments [29]. Using a limiting dilution analysis, TC-1 spheres were found to be significantly more tumorigenic in syngeneic mice compared to TC-1 adherent cells; P≤0.001 for P2, D1 cells and P = 0.0158 for P2, D7 cells (**Figure 6A**). D1 TC-1 spheres were more tumorigenic than D7 spheres; P = 0.04. TC-1 P2, D1 spheres were estimated to have a CIC frequency of 1 cell per 9,533 cells. P2, D7 spheres were the next most tumorigenic with an estimated CIC frequency of 1 cell per 21,918 cells, and adherent cells were found to be the least tumorigenic with an estimated frequency of 1 cell per 62,129 cells. For LLC cells implanted into syngeneic mice, there was a trend for lower CIC frequency amongst spheres (D1 cells, 1 per 29,694 cells and D7 cell 1 per 35,949 cells) compare to adherent cells (HM-LLC 1 per 46,989 cells and LM-LLC 1 per 49,686 cells), but this trend did not reach significance (**Figure 6B**). In NSG mice all dilutions of each cell line and culture condition tested was capable of forming tumors in 100% of mice. When tested, less than 500 cells for TC-1 and less than 1000 cells for LLC cells were sufficient to establish tumor in NSG mice (**Figure 6A and 6B**).

## Discussion

The majority of studies of CICs have used patient-derived material or established human tumor cell lines inoculated into immunocompromised mice. While these xenograft studies allow the identification of a sub-population of cells able to recapitulate a tumor in an immunocompromised mouse, they may not present an accurate picture of the characteristics of true CICs. In order to form a tumor in a human, potential CICs must interact with the immune system to prevent tumor recognition and elimination. As evidence for this, we showed that syngeneic immunocompetent mice require significantly more cells to initiate a tumor than immunocompromised mice. From this, it can be posited that there is a larger population of cells able to grow tumors in immunocompromised mice that cannot initiate tumors in their syngeneic counterparts, and that the true CICs may represent a much rarer population than determined in xenograph models.

The application of the sphere culture for detecting putative CICs has been used for various tumors [10], [16], [17], [30]. To our knowledge, this is the first time that a panel of murine lung cancer cell lines has been systematically examined for sphere forming capacity and enrichment of CICs (**Table 1**). In particular, TC-1 and HM-LLC spheres were capable of sustained growth in culture. At passage, a substantial number of cells die within the first 24 hours. This indicates that within sphere cultures there exists a sub-population of cells capable of self-renewal and proliferation, putative CICs. However, not all cell lines were capable of sustained growth as spheres. Seven out of the nine cell lines that formed spheres and were tested for long term culture capacity had a FE below or within a standard deviation of 1 (**Table 1**). A FE below 1 indicates that other cells with a limited proliferation capacity besides true CICs might form spheres, or that true CICs might asymmetrically divide over time into differentiated cells not capable of culture in sphere conditions. Hence, sphere formation over one or two passages is not as useful for identifying CICs as is prolonged sphere culture over several passages and the application of a mathematical model for assessing CIC symmetric division frequency [17].

In contrast to parental LLC and LM-LLC cells, HM-LLC cells are capable of forming spheres that could be passaged greater than eight times, and exhibited a robust fold expansion and symmetric division frequency. Additionally, sphere formation was noted for the metastatic murine lung adenocarcinoma lines 344P and 344SQ, but not for the non-metastatic cell line 393P. These three cell lines have been previously reported to form three-dimensional spheres in matrigel culture and the metastasis-prone lines have been characterized as capable of undergoing an epithelial-to-mesenchymal transition (EMT) [24]. It has been suggested that there may be a direct link between the EMT of cells and the acquisition of a stem cell-like/CIC phenotype [31]. Our results suggest that metastatic cells have an enriched ability to culture as non-adherent spheroids, and that metastatic cells are enriched for CICs. Future studies are needed to address this.

We demonstrated that the frequency of CICs changes over the course of a passage for murine lung cancer tumorspheres. Spheres represent a mixture of different cell types along the spectrum from CICs to short-term proliferating cells to differentiated cells [17]. At the time of passage for a fully formed multicellular sphere, cell-cell attachment is disrupted and a portion of these cells die within the first 24 hours. The surviving single cells go on to renew multicellular spheres in subsequent passages. As a result, at D1, there is a greater frequency of mitogen-responsive CICs in the culture than there is at D7. This indicates that the frequency of CICs to differentiated cells is not influenced so much by early passage versus late passage as it is by the time since passage (D1 versus D7). We assessed same passage (P2) TC-1 and HM-LLC D1 and D7 cells in comparison to adherent cells for tumorigenicity and phenotype of well-characterized CIC attributes including Hoechst 33342 dye SP [11], [32], [33], Aldefluor activity [12], Oct-4 [9], CD133 [8]–[10], [13], [34], and CD44 expression [13].

We found that the SP, Aldefluor activity, and CD133 expression were enhanced in HM-LLC spheres, but not TC-1 spheres compared to adherent cells. This calls into question reliance on the expression of a single putative antigen for identification of CICs for all tumor types and cell lines. We found that CD44 expression was decreased in sphere culture for both TC-1 and HM-LLC spheres compared to adherent cells. HM-LLC cells and parental LLC adherent cells expressed greater amounts of CD44 than LM-LLC cells. Expression of CD44, a cell-surface glycoprotein receptor involved in cell–cell interactions, adhesion and migration has been linked to increased metastasis [35]. CD44 expression did not have an effect on sphere-forming capacity as parental LLC cells could not form spheres. CD44 expression also did not have an effect on tumorigenicity as both LM and HM-LLC adherent cells formed tumors at similar frequencies. Spheres consisted of cells that expressed high, intermediate, and low amounts of CD44. We also noted that CD44 expression decreased between days 1 and 7 for HM-LLC spheres. This might indicate that D1 cells, enriched for the frequency of CICs, expressed more CD44 than D7 cells, when the sphere was fully formed and composed of a larger fraction of differentiated cells. The opposite was noticed for TC-1 spheres. We observed an increase in expression of Oct-4 in spheres of both TC-1 and HM-LLC compared to adherent cells. Oct-4 has been implicated in the self-renewal of undifferentiated embryonic stem cells [36] and other undifferentiated cells. Our findings suggest that sphere culture, without the need to isolate a particular cell population, enriches for cells that have an enhanced self-renewal capacity, a hallmark of CICs.

*In vitro* demonstration of CIC functional and phenotypic traits was confirmed with *in vivo* tumor initiation assays. We demonstrated that sphere culture enriched for more tumorigenic cells than adherent culture in syngeneic mouse models. TC-1 sphere-derived cells isolated on D1 were found to be more tumorigenic than adherent cells or D7 sphere-derived cells. This finding lends credence to the use of the sphere model for CIC enrichment, particularly in the absence of a definitive phenotypic marker. We found immunocompromised mice to be a less satisfactory model for studying murine lung CICs. Tumor take in the profoundly immunocompromised NSG mice was 100% for all culture conditions and dilutions studied, suggesting that virtually all the tumor cells have tumor-initiating capacity in this milieu. Different strains of immunocompromised mice exhibit differing levels and types of residual immune effector cells, and this in turn may alter the efficiency of tumor cell engraftment [37]. Additionally, the immunocompromised mouse microenvironment does not recapitulate the microenvironment in a human patient with naturally occurring cancer. Suggestions have been made that studies investigating the ability of different subtypes of cells to grow in immunocompromised mice does not so much distinguish CICs from non-CICs, as it demonstrates selection for cells that can best adapt to growth in murine tissue, and therefore might not represent a true approximation of CICs [29]. Alternatively, immunocompetent syngeneic models allow for interactions of the recipient mouse host immune system, a situation that more closely models cancer in humans. We propose that, where possible, syngeneic immunocompetent mouse models should be used for CIC studies.

As a result of heterogeneity both within a tumor [38] and within a population of patients, it is likely that even among lung tumors of the same type not all CICs are genotypically similar. Several markers have been proposed to identify CICs, but not all are uniformly expressed in all putative CICs. Barring identification of definitive lung CIC antigens, the tumorsphere assay is useful to enrich and characterize CICs. We demonstrated that there are differences in the frequency of CICs within TC-1 and HM-LLC tumorspheres over a single passage with D1 cells enriched in CICs compared to D7 cells. The highly clonal nature of these mouse lung cancer cell lines, their high passage number, lack of heterogeneity, and the fact that these are not primary tumor cells may suggest that the frequency of CICs may be over represented in these cell lines as compared to primary tumors. Studies of CICs in primary murine lung tumors derived from transgenic mice are in progress and should give more accurate representation of the numbers of CICs in murine tumors.

We have shown that tumorspheres from TC-1 and HM-LLC cell lines are enriched for functional and phenotypic identifiers of CICs including ALDH1, Oct-4, Hoechst 33342 SP, CD44 and CD133 expression, and self-renewal capacity. Importantly, sphere-derived cells exhibit greater tumorigenicity in syngeneic animal than adherent cells. Tumor formation in immunocompromised mice was seen at a lower initiating cell frequency than in syngeneic mice. Our studies suggest that in order to examine the true CICs, cells with the immune modulating properties, a syngeneic mouse model is needed. Further, the syngeneic models described in our study will allow the examination of CIC-derived cancer vaccines and immunotherapy, treatment that are unable to be assessed in immunocompromised animals.
